# Preoperative hydronephrosis is a predictive factor of ureteral stenosis after flexible ureteroscopy: a propensity scores matching analysis

**DOI:** 10.1186/s12894-021-00917-1

**Published:** 2021-11-11

**Authors:** Yuefan Shen, Anping Xiang, Sihai Shao

**Affiliations:** Department of Urology, The First People’s Hospital of Huzhou, No.158 Guangchanghou Road, Huzhou, Zhejiang Province China

**Keywords:** Ureteral stenosis, Flexible ureteroscopy, Upper ureteral stones, Hydronephrosis, Propensity scores matching analysis

## Abstract

**Objectives:**

Ureteral stenosis is a serious complication of flexible ureteroscopy. How to predict the possibility of stricture before surgery is an important topic. This research retrospectively studied the influence of preoperative hydronephrosis on ureteral stenosis after flexible ureteroscopy, to explore whether the preoperative hydronephrosis could predict postoperative ureteral stenosis.

**Methods:**

We conducted a retrospective study on patients who received flexible ureteroscopy in our hospital for upper ureteral calculi from January 2015 to June 2018. Patients were followed-up for 36 months after surgery, and intraoperative and postoperative complications were recorded. We divided patients into the mild hydronephrosis group and moderate to severe hydronephrosis group. Preoperative clinical baseline data of the patients were adjusted by propensity matching score analysis. Differences of intraoperative ureteral injury, operative time, postoperative ureteral stricture, and SFR one month after surgery was statistically analyzed. Kaplan–Meier’s method and Log-rank test were used to compare the differences in the cumulative incidence of ureteral stenosis between the two groups. Cox regression was used to compare the hazard ratio of ureteral stenosis between the two groups.

**Results:**

A total of 447 patients with 469 sides surgery were included, including 349 sides in the mild hydronephrosis group and 120 sides in the moderate to severe hydronephrosis group. Twenty-nine patients with 30 sides developed ureteral stenosis. Before and after propensity, the incidence of ureteral stricture matching analysis was 6.4% and 8%, respectively. There were statistical differences in ureteral stricture and injury, but the statistical differences in SFR and operation time were inconsistent. Kaplan–Meier showed a significant difference in the cumulative incidence of ureteral stenosis between the two groups.

**Conclusions:**

Patients with moderate to severe hydronephrosis before surgery were more likely to have an intraoperative ureteral injury and postoperative ureteral stricture after FRUS. Preoperative hydronephrosis is an important predictor of ureteral stricture.

## Background

With the development of the flexible ureteroscope technical, the FRUS has gradually become an choice for upper ureteral calculi. Guidelines of EAU [1] suggested that "percutaneous nephrolithotomy is considered for ureteral stone larger than 15 mm with stone impaction." However, in practical work, the indications for flexible ureteroscope are gradually relaxed. Some doctors prefer a flexible ureteroscope because it is more minimally invasive. The FRUS presents several advantages over percutaneous nephrolithotomy, including reduced post-operational pain, lower risk of hemorrhage and shorter hospitalization time. It is more acceptable to patients. Nevertheless, further minimally invasive does not always mean better. As the number of FRUS increased, so did the complication of surgeries. Ureteral stenosis is a rare and severe complication after FRUS. It eventually requires reoperation, causing physical pain and financial burden to the patients. Early ureteroscopy studies reported ureteral stricture rates around 0.30%-23.81%, mostly in rigid and semi-rigid ureteroscopy studies [2]. Some scholars have studied the risk factors of ureteral stenosis, and believe that stone burden, stone impaction and operation time are important factors [3]. "Stone impaction" has been mentioned in the EAU guideline and many other literatures [1, 4, 5]. In recent years, research has shown that thickness of the renal parenchyma and ureteral wall on CT are related to stone impaction [6, 7]. In addition, few studies have investigated how to judge "stone impaction." In clinical diagnosis and treatment, we find that the degree of hydronephrosis is closely related to ureteral injury and stricture irrespective of the stone size. The degree of hydronephrosis may represent the severity of stone impaction, which may be another predicator for ureteral stricture. We undertake a retrospective study to evaluate this hypothesis.

## Methods

### Patients

From January 2015 to June 2018, 688 patients underwent flexible ureteroscopy for upper ureteral stone treatment at our institution. Most of them were patients with high stone burden unsuitable for SWL, or patients with ureteral calculi before unsuccessful SWL. The clinical data were obtained by reviewing medical records. The inclusion and exclusion criteria were like following.

Inclusion criteria: (1) patients with upper ureteral calculi above the level of iliac vessels, including calculi at the ureteropelvic junction; (2) double J tubes were placed one to two weeks before surgery.

Exclusion criteria: (1) previous history of ipsilateral upper ureteral calculi surgery [8], including ureteroscopy, percutaneous nephrolithotomy, laparoscopy or open surgery; (2) preoperative ureteral stricture; (3) patients with too much missing data; (4) urinary malformation; (5) neurogenic bladder; (6) orthotopic neobladder history.

We selected patients with mild hydronephrosis as the case group, and patients with moderate to severe hydronephrosis as the control group. The primary outcome was ureteral stenosis. The secondary outcome was operative time, intraoperative ureteral injury and stone clearance rate one month after surgery.

### Definition of terms in this paper

Onset time: the onset time was days from the onset of symptoms until the date of surgery.

Calculi size and hydronephrosis: these values were obtained from the CTU. The width of the renal pelvis less than 2 cm was considered mild hydronephrosis, while that larger than 2 cm was considered moderate to severe hydronephrosis.

Stone burden calculation: maximum length × maximum width × π × 0.25(π = 3.14).

Stone removal: KUB indicates residual stone ≤ 4 mm.

### FRUS procedure

All the patients finished CTU before operation to exclude ureteral stenosis. Double J tubes were placed preoperatively for one to two weeks to dilate the ureter. All patients received general anesthesia. The surgeon used a Ureteroscope (Wolf 6/7.5 or 8/9.8) to remove the stent, and then found the ureteral orifice. A guidewire was introduced via which a rigid ureteroscope was passed through the ureteral orifice. The middle and lower sections of the ureter were explored by a ureteroscope. After the endoscope was removed, an access sheath (F12, Cook Company) was placed into the ureter along the guide wire, and a flexible ureteroscope (8.5/9.9F, Olympus) was placed at the inferior end of the stones. The stones were fragmented by 200 μm Holmium laser with power of 0.8–1.2 J and frequency of 15–25Hz. Postoperatively, an F6 Double-J stent was placed routinely. Antibiotic was used to prevent infection for 24–48 h. The Double J tube was removed 15–60 days later according to the situation.

### Postoperative follow-up

All patients were followed up in the outpatient department. They underwent KUB one month after surgery, and B-ultrasound of the urinary system three months, six months, one year, two years and three years after Double J tube removed. If there was no aggravation of hydronephrosis, we defined it as no ureteral stenosis. If B-ultrasound indicated gradual aggravation of hydronephrosis, CTU would be performed to determine the cause of obstruction.

### Postoperative complications

Ureteral injury was classified according to Ureteroscopic Lesion Scale (PULS) [9]. Postoperative complications were graded according to the Clavien–Dindo grading system [10].

### Statistical analysis

Data were collected retrospectively, entered into an Excel 16 software, and analyzed with the IBM SPSS 25. The independent sample *t*-test (continuous variable) or chi-square test (categorical variable) were used for comparing surgical outcomes and characteristics of the two groups. The clinical baseline characteristics of patients were adjusted by 1:1 propensity matching. After propensity scores matching analysis, paired sample T test (continuous variable) or McNEMA test (categorical variable) were adopted to compare the clinical characteristics of the two groups. The Kaplan–Meier method was applied to estimate cumulative incidence, and log-rank test was constructed to compare the differences between the two groups. A Cox proportional-hazards model was used to evaluate the hazard ratio for ureteral stricture in both groups.

## Results

**Fig. 1:**
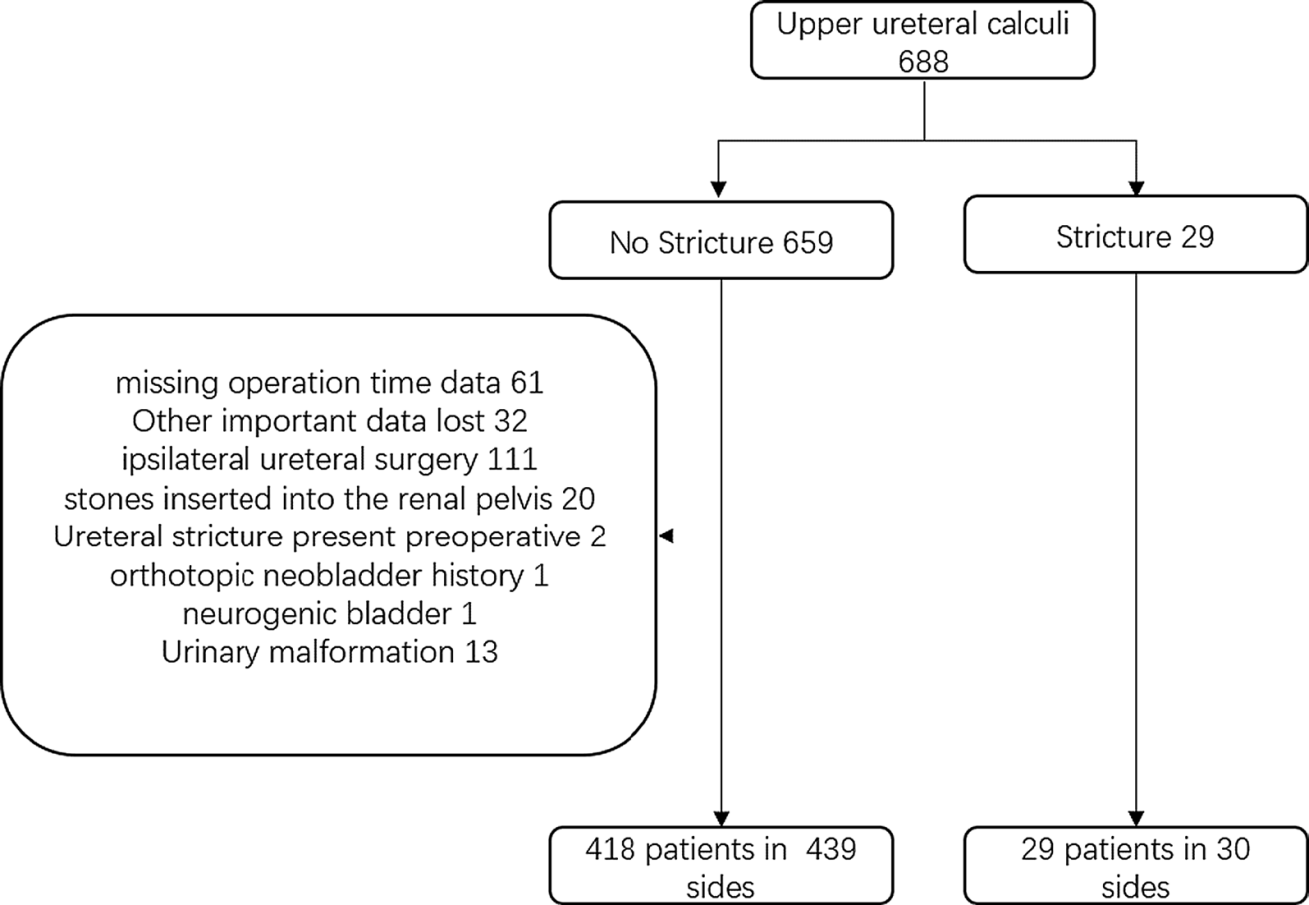
Patient selection

A total of 688 patients with upper ureteral calculi underwent flexible ureteroscope in our hospital. There were 659 cases of non-stricture and 29 cases of strictures. Sixty-one patients had been missing operation time data; Thirty-two patients had been missing other important data. The patients excluded for other reasons were shown in Fig. 1. A total number of 469 sides surgical procedures for 447 patients were included in our retrospective analysis. 349 sides were mild hydronephrosis, and 120 sides were moderate to severe hydronephrosis. The average age of patients was 50.43 ± 12.14 years, the mean time since onset was 46.80 ± 55.34 days, the mean BMI was 24.05 ± 3.65, and the mean stone burden was 86.66 ± 46.76 mm^2^. Before propensity matching, there were significant differences in age, onset time, renal colic and stone burden between the two groups. A total of 75 pairs of patients were obtained after 1:1 propensity matching, and all baseline characteristics were balanced between the two groups (Table 1). After surgery, twelve patients (13 sides of strictures) were lost to follow up, including 8 patients (9 sides of strictures) with confirmed stenosis. They were transferred to other hospitals. Data were not available for the remaining 4 patients with non-stenosis due to review at other hospitals. The differences of observational outcomes before and after propensity matching are shown in Table 2. A total of 29 patients had ureteral stenosis on 30 sides after operation. The stenosis rate before and after the propensity matching was 6.4% and 8%, respectively. There were significant differences before and after propensity matching. Although the SFR and operation time reached statistical significance before propensity matching, there were no clinically significant differences after propensity matching.

**Table 1:** Patient baseline characteristics

	Before propensity matching			After propensity matching		
	Mild hydronephrosis	Moderate and severe hydronephrosis	*P* value	Mild hydronephrosis	Moderate and severe hydronephrosis	*P* value
	N = 349	N = 120		N = 75	N = 75	
Age (year)	49.58 ± 12.03	52.91 ± 12.18	0.010	52.00 ± 10.54	53.29 ± 11.94	0.483
Male (%)	69.1%	69.2%	0.982	68.0%	66.7%	1.000
BMI	23.97 ± 3.53	24.27 ± 4.00	0.475	24.43 ± 3.21	24.26 ± 3.95	0.779
Disease time (days)	41.95 ± 45.41	60.88 ± 75.81	0.011	46.63 ± 42.26	43.00 ± 41.59	0.596
Renal colic (%)	65.6%	31.7%	0.000	34.7%	37.3%	0.804
Fever (%)	4.6%	7.5%	0.220	8.0%	8.0%	1.000
SWL (%)	20.9%	19.2%	0.682	24.0%	21.3%	0.815
Stone burden (mm^2^)	78.65 ± 41.93	109.89 ± 52.17	0.000	97.94 ± 39.36	99.75 ± 39.86	0.675

**Table 2:** Patient observational outcomes

	Before propensity matching			After propensity matching		
	Mild hydronephrosis	Moderate and severe hydronephrosis	*P* value	Mild hydronephrosis	Moderate and severe hydronephrosis	*P* value
	N = 349	N = 120		N = 75	N = 75	
Ureteral stenosis	2.3%	18.3%	0.000	0	16.0%	0.000
Ureteral injury (≥ grade2)	5.2%	42.5%	0.000	5.3%	37.3%	0.000
Operation time	67.75 ± 33.62	83.29 ± 37.89	.000	74.90 ± 31.29	80.12 ± 37.53	0.383
SFR	88.5%	78.3%	.006	77.3%	80.0%	0.839

**Fig. 2:**
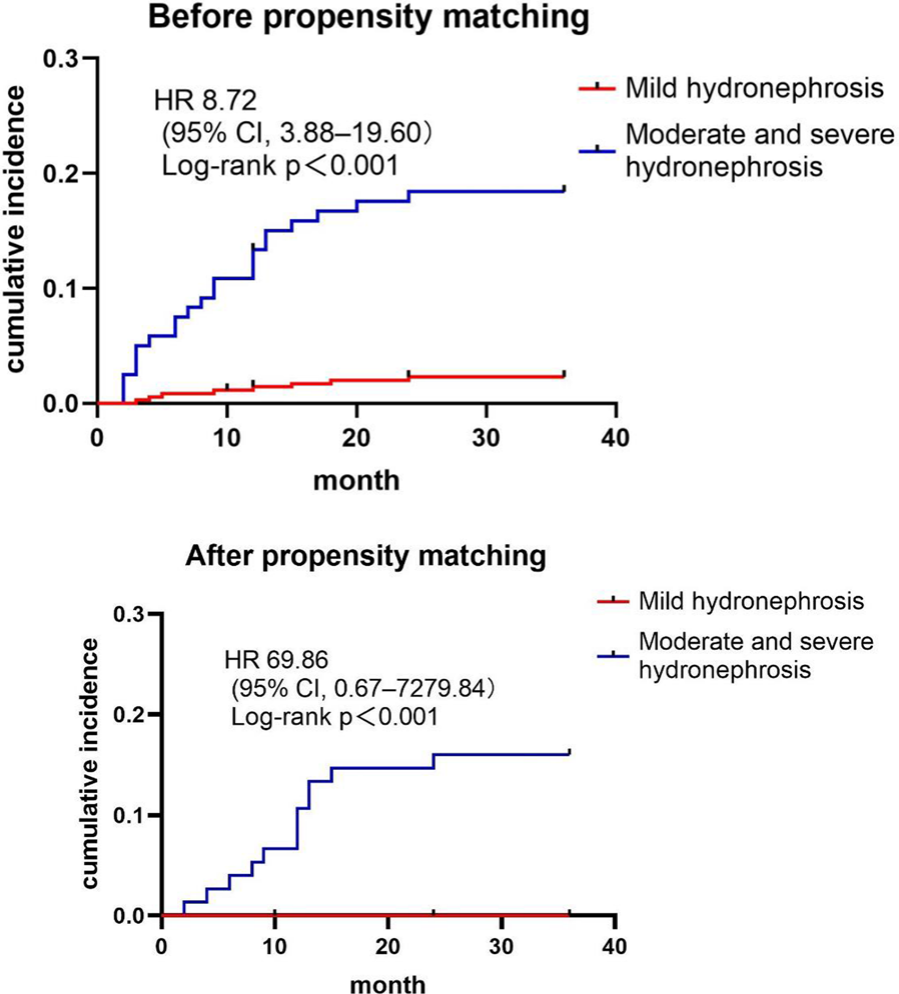
Kaplan–Meier cumulative event curves of ureteral stenosis before and after propensity score-matching. Adjusted risks of mild hydronephrosis relative to Moderate and severe hydronephrosis are shown

The median follow-up time of both groups was 36 months. The mean follow-up time of mild hydronephrosis group and moderate to severe hydronephrosis group was 35.26 ± 4.32 months and 30.88 ± 10.87 months, respectively. Figure 2 showed that the cumulative incidence of ureteral stenosis was higher in the moderate to severe hydronephrosis group before propensity matching [HR 8.72; 95% CI 3.88–19.60; *P* = 0.000]. When baseline clinical characteristics were matched, moderate to severe hydronephrosis group was associated with a significantly higher risk of ureteral stenosis (HR 69.86;95% CI 0.67–7279.84; *P* = 0.073).

The incidence of ureteral injury was higher in the moderate to severe hydronephrosis group, most of which were grade 1–2. There were only 4 cases of grade 3 injuries, among which 3 cases had ureteral strictures, and no injury above grade 4. Data for postoperative complications were collected. There were 2 cases of low fever (Clavien1), 12 cases of high fever (body temperature > 38.5) with urinary tract infection (Clavien2), 66 cases of residual stones (Clavien3a), 29 cases of ureteral stricture requiring reoperation (Clavien3b), and 1 case of postoperative sepsis requiring intensive care (Clavien 4). Long-term follow-up revealed 2 cases of asymptomatic severe hydronephrosis (Clavien3b).

Twenty-nine patients had postoperative stenosis, of which 21 underwent reoperation in our hospital. Two patients accepted nephrectomy due to severe hydronephrosis. Eleven patients were cured after partial ureterectomy and ureteral end-to-end anastomosis. One person was cured after ureteral balloon dilation. Four patients had ureteral laser incision, and after failure, they went to other hospitals for further treatment. Three patients were placed with ureteral stents, and one of them had ureteral recanalization after removal of the stents. The other two patients did not improve the hydronephrosis, so they went to other hospitals for further treatment.

## Discussion

The main results of our research were as follows. First, the incidence of stenosis in moderate to severe hydronephrosis group was higher than that in the mild hydronephrosis group. Second, in the moderate to severe hydronephrosis group, the incidence of intraoperative ureteral injury was higher; the duration of operation was longer, and the postoperative SFR was lower. However, there was no difference in the SFR and operation time between groups after adjusting for baseline. Third, the Kaplan–Meier cumulative event curve showed that incidence of ureteral stricture was higher in the moderate to severe hydronephrosis group.

There are several studies on the risk factors of ureteral stricture. In a prospective study, AMR E. Darwish et al. included variables such as stone burden, stone impaction, operative time, and double J tube placement time. Logistic regression analysis found that stone impaction was associated with ureteral stricture. There were only 4 cases of Ureteral stricture in the article [3]. Xeng Inn Fam et al. explored the risk factors for ureteral stricture after ureteroscopy in the treatment of impaction calculi. The variables included ureteral perforation, mucosal injury, stone impaction, impaction time, stone size, impaction location, etc., but they failed to find any predictive factors. However, there were only 5 cases of Ureteral stricture [11]. Both above two studies used Logistic regression analysis to correct other factors, but the sample size and the number of stenosis cases were small, which affected power and statistical analysis. In our study, 29 patients with ureteral stenosis were included. Patients were grouped according to hydronephrosis. We got 75 pairs of matched patients through propensity matching. Sample size was expanded as far as possible to improve the accuracy of statistical results.

There are many speculations about the mechanism of ureteral stenosis after Ureteroscopic surgery, such as stone impaction, access sheath injury, large stone burden, intraoperative ureteral perforation and the surgeon's experience [2, 12]. Daniel A Wollin et al. designed an in vitro model to study the water temperature at different perfusion rates during holmium laser lithotripsy. The authors found that, even with high laser power, adequate irrigation could still maintain a relatively stable temperature. With the decrease of the perfusion velocity, the laser-induced temperature could be significantly increased in spite of lower laser power. This condition might cause ureteral tissue damage [13]. Shiulian Chen et al. included eight studies with a total of 1760 patients in a meta-analysis. The results showed that the incidence of ureteral stenosis after holmium laser lithotripsy was higher than pneumatic lithotripsy. The authors believed that this issue demanded further research [14]. In actual practice, we find that if the calculi cannot be pushed during the operation, ureteral stricture is more likely to happen postoperatively. If the ureteral stone is pushed into the renal pelvis after a brief time holmium laser lithotripsy, there is almost no postoperative ureteral stenosis. This is consistent with Daniel A Wollin's model. The above studies show that stone impaction and ureteral injury may be causal. The key is how to predict stone impaction before surgery. Stone impaction is a vague concept, which is difficult to judge only by preoperative clinical manifestations. The thickening of the ureteral wall on CT may reveal stone impaction and the stone wrapping by polyps, which can predict postoperative stenosis [6]. In our practice, we found that the degree of hydronephrosis was associated with postoperative stenosis, which might be another indicator to predict postoperative stenosis after FRUS. This point was confirmed by our retrospective study.

Our study has some limitations. First, this is a single-center retrospective study with a limited sample size. Surgical equipment and techniques are restricted by the center. Randomized controlled studies will be ideal, but studies of postoperative complications may be difficult to achieve. Propensity matching can be an effective tool to reduce potential bias and baseline differences in a retrospective study. Second, after propensity scores matching analysis, there is no statistical difference of the operation time and SFR, possibly because the patients with ipsilateral ureteral calculi and large renal stones account for a sizeable proportion, which prolongs the operative time and reduces SFR. However, an extended version of exclusion criteria may include a relatively small number of patients, which may reduce the strength of the conclusions in this paper. Third, the author did not participate in all the operations and could only evaluate the patient's condition according to the medical records and imaging. So intraoperative details may be omitted. Fourth, Patients are lost to follow-up and the missing data may affect our results.

## Conclusions

Preoperative moderate to severe hydronephrosis is a predictor of postoperative ureteral stricture. Patients with moderate to severe hydronephrosis have an increased risk of complications such as ureteral injury and postoperative stricture after FURS. The prevalence of ureteral stricture has increased over time. Clinicians may choose the appropriate surgical procedure based on the preoperative degree of hydronephrosis.

## Data Availability

The datasets used and/or analysed during the current study are available from the corresponding author on reasonable request.

## Abbreviations

FURS: Flexible ureteroscopy
SWL: Extracorporeal shock wave lithotripsy
BMI: Body mass index
CTU: Computed tomography urography
KUB: X-ray of kidney ureter bladder
SFR: Stone-free rate
HR: Hazard ratio
CI: 95% Confidence interval
